# Supplementary material for: A Likelihood Approach for Real-Time Calibration of Stochastic Compartmental Epidemic Models
Source: PLoS Comput Biol. 2017 Jan 17;13(1):e1005257. doi: 10.1371/journal.pcbi.1005257 (PMC5240920; doi:10.1371/journal.pcbi.1005257)
Supplement: S1 File — (TAR.GZ) [file pcbi.1005257.s014.tar.gz › HSPH_Online-SI-Revision/Additions/EnKF08/n100-extreme-SEITR_noisy/n100-extreme-SEITR_noisy_PF2_EnKF-summary2.pdf]

peak -8	3w spec	3w cum	5w spec	5w cum	end	3w spec LOG	3w cum LOG	5w spec LOG	5w cum LOG	end LOG
mean	2001.	8804.3	106.1	798.3	52.5	1.1	1.4	1.3	0.8	0.5
median	737.8	4136.3	92.1	565.2	46.	1.1	1.5	1.2	0.8	0.4
quantile .1	101.	162.1	70.8	94.9	32.	0.6	0.4	0.6	0.5	0.2
quantile .9	5721.6	25 391.8	164.6	1709.3	89.	1.6	2.3	2.2	0.5	1.2
25	8	23	11	36						

peak -4	3w spec	3w cum	5w spec	5w cum	end	3w spec LOG	3w cum LOG	5w spec LOG	5w cum LOG	end LOG
mean	87.7	89.7	95.2	77.9	78.6	1.8	0.8	2.6	0.9	1.1
median	93.1	87.7	95.8	87.	87.3	1.8	0.8	2.8	1.1	1.2
quantile .1	66.5	71.3	90.3	58.3	55.3	0.6	0.3	1.4	0.4	0.4
quantile .9	96.7	105.3	99.2	91.1	91.7	2.6	1.2	3.5	0.4	1.6
5	26	0	20	15						

peak	3w spec	3w cum	5w spec	5w cum	end	3w spec LOG	3w cum LOG	5w spec LOG	5w cum LOG	end LOG
mean	90.6	88.5	94.5	87.5	87.	1.3	1.	1.6	1.	1.
median	91.	89.6	94.9	88.5	87.8	1.2	0.9	1.7	0.9	0.9
quantile .1	79.4	80.2	83.2	77.1	76.5	0.7	0.6	0.8	0.6	0.6
quantile .9	99.2	94.8	105.	94.5	95.2	2.	1.4	2.4	0.6	1.5
16	19	8	21	24						

peak +4	3w spec	3w cum	5w spec	5w cum	end	3w spec LOG	3w cum LOG	5w spec LOG	5w cum LOG	end LOG
mean	186.7	195.3	294.9	189.7	193.	0.8	0.4	1.1	0.4	0.4
median	125.3	159.9	111.3	141.6	135.2	0.6	0.4	0.9	0.4	0.4
quantile .1	76.3	82.4	86.3	82.9	86.9	0.3	0.3	0.5	0.3	0.3
quantile .9	354.	348.3	550.5	355.	349.9	1.6	0.6	2.	0.3	0.6
45	46	39	45	45						

peak +8	3w spec	3w cum	5w spec	5w cum	end	3w spec LOG	3w cum LOG	5w spec LOG	5w cum LOG	end LOG
mean	4810.8	2742.	15 549.6	2833.9	3025.3	1.	0.8	1.2	0.8	0.9
median	984.3	1115.8	990.	1144.8	1159.4	0.9	0.7	1.1	0.7	0.8
quantile .1	241.9	305.3	212.1	251.2	231.	0.5	0.4	0.7	0.4	0.4
quantile .9	9845.	8966.8	19 372.	8397.8	7998.6	1.6	1.4	1.9	0.4	1.4
40	22	44	22	23	{}	{}	{}	{}	{}	{}
